# The Optimal Tetralogy of Fallot Repair Using Generative Adversarial Networks

**DOI:** 10.3389/fphys.2021.613330

**Published:** 2021-02-23

**Authors:** Guangming Zhang, Yujie Mao, Mingliang Li, Li Peng, Yunfei Ling, Xiaobo Zhou

**Affiliations:** ^1^West China Biomedical Big Data Center, West China Hospital, Sichuan University, Chengdu, China; ^2^Department of Cardiovascular Surgery, West China Hospital, Sichuan University, Chengdu, China; ^3^School of Biomedical Informatics, The University of Texas Health Science Center at Houston, Houston, TX, United States

**Keywords:** Tetralogy of Fallot, generative adversarial networks, pulmonary artery, patch, cardiac CT

## Abstract

**Background:**

Tetralogy of Fallot (TOF) is a type of congenital cardiac disease with pulmonary artery (PA) stenosis being the most common defect. Repair surgery needs an appropriate patch to enlarge the narrowed artery from the right ventricular (RV) to the PA.

**Methods:**

In this work, we proposed a generative adversarial networks (GANs) based method to optimize the patch size, shape, and location. Firstly, we built the 3D PA of patients by segmentation from cardiac computed tomography angiography. After that, normal and stenotic areas of each PA were detected and labeled into two sub-images groups. Then a GAN was trained based on these sub-images. Finally, an optimal prediction model was utilized to repair the PA with patch augmentation in the new patient.

**Results:**

The fivefold cross-validation (CV) was performed for optimal patch prediction based on GANs in the repair of TOF and the CV accuracy was 93.33%, followed by the clinical outcome. This showed that the GAN model has a significant advantage in finding the best balance point of patch optimization.

**Conclusion:**

This approach has the potential to reduce the intraoperative misjudgment rate, thereby providing a detailed surgical plan in patients with TOF.

## Introduction

Tetralogy of Fallot (TOF) is a type of congenital cardiac disease affecting around 3%oo individuals (Apitz et al., 2009; Khan et al., 2019). Four typical defects of TOF comprise pulmonary stenosis, ventricular septal defect, right ventricular (RV) hypertrophy, and overriding aorta. With oxygen-poor blood induced by TOF cycling over the body, faint, dyspnea, and cyanosis may occur (Kalra et al., 2010; Chiu et al., 2012). The repair of TOF includes closure of the ventricular septal defect and ensures the unobstructed blood flow from ventricle to aorta. This repair is usually done several months after birth. When pulmonary stenosis presents, a patch across the RV outflow tract is implanted to mitigate pulmonary regurgitation. Meanwhile, a volume load is exerted on the RV, which influence RV function both at rest and at exercise and correlate with the degree of PR. Treatment for PR would increase the exercise tolerance of individual.

Currently, we can accurately diagnose the TOF based on cardiac computed tomography (CTs). However, we are unable to accurately predict the patch size, shape, and location following the virtual repair surgery despite of multiple approaches for trial. It is an urgent need, both from doctors and patients, to develop a reliable patch simulation tool to accurately plan the repair surgery of TOF. To this end, we proposed a predicting system that can accurately simulate the patch size, shape, and location for repair of TOF.

Through integrating and utilizing multiple network architectures, deep learning is capable of meticulous image processing, such as segmentation, object detection, image fusion, and classification. Unlike conventional machine learning that lean on feature extraction for training algorithms, deep networks allow deep learning for direct image data proceeding. Up to today, deep learning has been applied in many different research fields to solve complicated problems (Litjens et al., 2017), made possible through parallel computing and big datasets. Acquiring a large annotated medical imaging dataset can be rather challenging for classification problems (e.g., discriminating healthy and diseased subjects), as one training example then corresponds to one subject. Data augmentation, e.g., rotation, cropping, and scaling, is normally used to increase the amount of training data, but can only provide limited alternative data. A more advanced data augmentation technique, generative adversarial networks (GANs) (Goodfellow et al., 2014), uses two competing convolutional neural networks (CNNs): one that generates new samples from noise and one that that discriminates samples as real or synthetic. The most obvious application of a GAN in medical imaging is to generate additional realistic training data in order to improve classification performance. Another application is to use GANs for image translation, e.g., to generate CT data from magnetic resonance (MR) images or vice versa (Dar et al., 2019). This can for example be very useful for multimodal classification of healthy and diseased subjects, where several types of medical images (e.g., CT and MRI) are combined to improve sensitivity. In medical image analysis, GANs contribute to mitigate the confines of dataset sizes and annotation (Karras et al., 2020; Yang et al., 2020). For example, to better classify liver lesion in CNN, synthetic CT images are generated using conditional GANs (Frid-Adar et al., 2018) and introduced into training for data augmentation. In terms of accuracy, GAN based data augmentation (Madani et al., 2018) outperform the traditional one in classification of chest X-ray. While synthetic images generated by GANs are visually pleasing, they may not always provide meaningful features to ameliorate the performance of model for task solving. The GANs provide an appropriate way to learn deep representations without widespread use of labeled training data.

In order to simulate an appropriate patch to expand the narrowed pathway from the RV to the pulmonary artery (PA). In this work, we proposed a GAN based method to optimize the patch size, shape, and location. Firstly, all CT images were resliced into view parallel to the cross-section of the artery. Three-dimensional geometric models were constructed based on the cardiac CT angiography. After that, both stenotic and normal area on PA were detected on each patient based on 3D models. Here, area with diameter less than 5 mm is identified as stenotic area, and a normal area with equal length is also determined on the same PA. These detected areas were then labeled back on the CT images and formed two sets of sub-images. Next, a GAN was trained based on these sub-images. Finally, fivefold cross-validation (CV) was performed to validate our model.

## Methodology

### Theoretical Preliminaries

Generative adversarial network is a deep learning method that can be characterized by training a pair of networks in competition with each other. As a deep generative neural-network architecture, GAN consists of two sub-networks: the generator *G*, and the discriminator *D*. The generator learns to map from a latent space *s* to a data distribution of interest *y* in a target domain, and discriminator learns to distinguish candidates produced by generated image *G*(*z*) from the true image data *m* (Goodfellow et al., 2014). The aim of training the generative network is to increase the discriminative error rate of the discriminator network, generating images that are indistinguishable from the real data, and the discriminator may be characterized as a mapping function transferring image data to a probability, converting the image from the real data distribution to apart generated and real images. To implement this, GANs use the following adversarial loss function (FGAN):

(1) $$F_{G\,A\,N}\,\left(G,\,D\right)=V\,m\,\left[\text{log}\,D\,\left(m\right)\right]+V\,s\,\left[\text{log}\,\left(1-D\,\left(G\,\left(s\right)\right)\right)\right],$$

where *V* represents expected value for the training. The generator *G* tries to minimize the adversarial loss, while the discriminator *D* tries to maximize the adversarial loss. This competitive process improves modeling the networks until the counterfeits are indistinguishable from the genuine one. When the networks converge, the generator *G* is able to produce realistic fake image data that the discriminator *D* cannot distinguish (Goodfellow et al., 2014). For the purpose of stabilizing the training process, a squared loss can replace the negative log-likelihood cost for adversarial loss in (1):

(2) $$F_{G\,A\,N}\,\left(D,G\right)=-V\,m\,\left[{\left(D\,\left(m\right)-\,1\right)}^{2}\right]-V\,s\,\left[D\,{\left(G\,\left(s\right)\right)}^{2}\right]$$

In our study, 3D geometric models of the PA were constructed by segmenting the cardiac CT angiography. Both stenotic and normal area on PA were detected and labeled. Then, sub-images of the stenotic and normal area were served as input data to train the GAN model.

**FIGURE 1:**
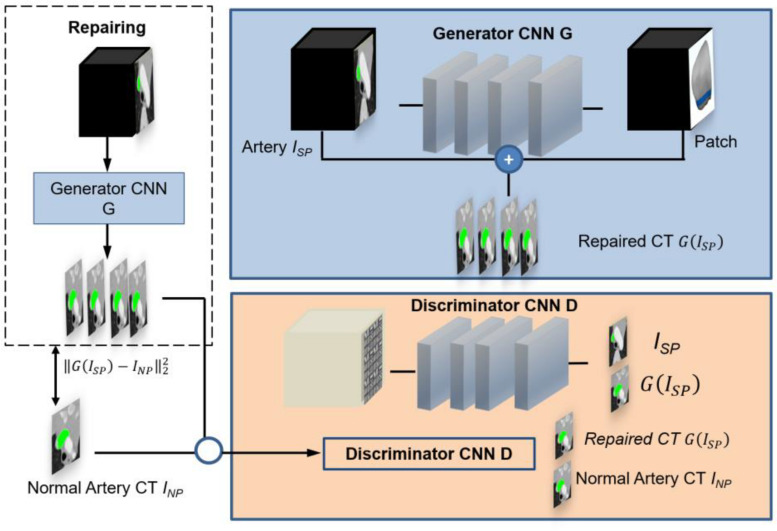
Overview of the pipeline for simulating the optimal patch design based on pulmonary artery CT. The GAN comprises a generator CNN *G* and a discriminator CNN *D*. Regression is used to determine the pulmonary artery value at every voxel in a pulmonary artery stenosis CT. This is done by a skip connection which augments an estimated patch from hemodynamics analysis to the input stenosis CT image. The discriminator is trained to discriminate repaired CT images from normal pulmonary artery CT images.

Figure 1 shows the GANs system for optimal patch design, which consists of two structural components. One component has a CNN generator *G* for analyzing the stenotic area *I*_*SP*_ of the PA on CT. The generator denoted by *G*(*I*_*SP*_) represents an estimation for the repaired pulmonary CT images of *I*_*NP*_. The networks use two methods to calculate resemblance between *G*(*I*_*SP*_) and *I*_*NP*_. Firstly, if voxels in *I*_*SP*_ and *I*_*NP*_ are consistent from each other, the error between generator *G*(*I*_*SP*_) and *I*_*NP*_ is minimized during training. Secondly, the CNN discriminator *D* will be trained to differentiate between *G*(*I*_*SP*_) and *I*_*NP*_ simultaneously. When the discriminator *D* can recognize the distinction simply, such as the generated CT images do not seem like normal pulmonary CT images, the generator needs to improve its approximation. As a result, both generator *G* and discriminator *D* networks have different missions in the training. The generator *G* implements a regression of voxel data of normal PA CT and the discriminator *D* performs classification of normal and pulmonary stenosis artery CT.

### Generator CNN

The PA stenosis CT image *I*_*SP*_ is transformed by the generator CNN *G* into an repaired image *G*(*I*_*SP*_) approximating the reference normal pulmonary CT image *I*_*NP*_. We assume that *I_*NP*_* = *I*_*SP*_ + *P*, where *P* represents an extensional patch used to repair the stenosis artery. Hence, the deep learning layers in the CNN has the mission to estimate the size and location of patch *P* by mimicking the normal area of PA. The extensional patch *P* is predefined as a rectangle patch with equal length of the stenotic.

In our networks, the CNN generator contains a 3D rectangular volume of PA voxels. It usually consists of four consecutive convolution layers with the convolution kernels for calculation (Wolterink et al., 2017). The final convolution layer brings out the estimated optimal patch through a linear activation function in networks. This patch is then calculated from the normal pulmonary CT image to a stenosis CT image *G*(*I*_*SP*_). The trained layers exceeded the final convolution layer apply a leaky rectified linear activation functions (LReLUs) to make the training progress stably. In this model, the weights are initialized with a normal distribution (μ = 0, σ = 0.01). In the CNN generator, batch normalization (Yuan et al., 2019) is using re-centering and re-scaling method to make the GANs faster and more stable by normalization of the input layer. The mini-batch normalization can reduce the number of training epochs required for deep networks.

### Discriminator CNN

The discriminator takes either a normal pulmonary CT sub-image *I*_*NP*_ or a processed repaired CT sub-image G(*I*_*SP*_) as input data, it distinguishes whether the input image is a normal pulmonary CT image or not. The input data to the discriminator is a 3D rectangular volume box. Convolution layers are organized in four blocks. Like the generator, LReLU activation functions and batch normalization are applied in discriminator *D*. A sigmoid activation to determine whether the input is a normal pulmonary CT image (label 1) or not (label 0) is employed in the final layer contains. The weights in the discriminator *D* are initialized by the Adam optimizer (Kingma and Ba, 2014), which usually be used replace the stochastic approximation of gradient descent optimization procedure to update GANs weights iteratively in the training procedure.

## Experiments and Results

Eighteen male and twelve female TOF patients who underwent repair surgery at an average age of 1.8 months, ranging from 1 to 3.5 months, were randomly chosen and included into the study. CT angiography images of each patient were used for pulmonary segmentation. Multi-slice CT of the stenosis pulmonary were taken at peak diastole before operation and imported into Mimics 19.0 Image Software (Materialise, Leuven, Belgium) for further processing. Three-dimensional anatomical models of PA were reconstructed at a threshold level of 320–800 Hounsfield, which allowed for the separation of main PA, left PA and right artery and preserved the detailed geometry features in the meantime.

**FIGURE 2:**
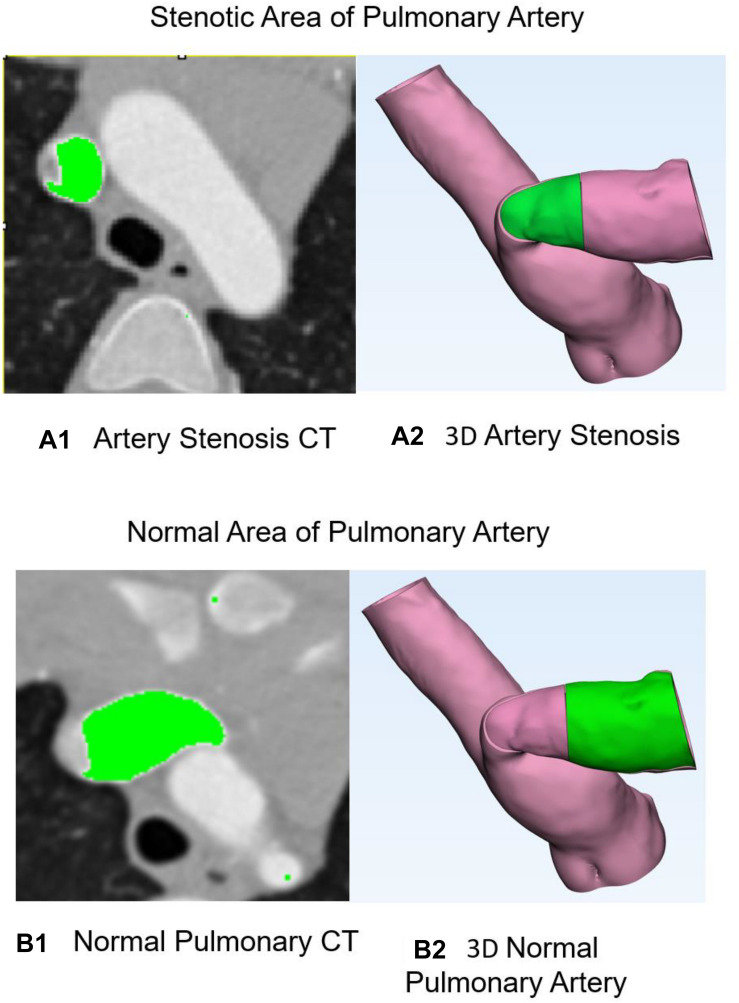
Stenosis and normal pulmonary artery. **(A1)** CT of pulmonary artery stenosis where the stenosis part of artery is marked in green. **(A2)** Three-dimensional artery with stenosis part in green. **(B1)** CT of pulmonary artery stenosis where the normal part of artery is marked in green. **(B2)** Three-dimensional artery with normal part in green.

Figure 2 shows the training data of stenosis and normal PA. Figure 2A1 demonstrates the CT of PA stenosis where the stenotic part of artery is marked in green. Figure 2A2 shows the 3D artery with stenosis part in green. Figure 2B1 demonstrates the CT of PA stenosis where the normal (without stenosis) part of artery (which is wider than that in Figure 2A1) is marked in green. Figure 2B2 shows the 3D artery with normal part in green. To limit the region-of-interest and reduce computational complexity, we restricted the zone to the 10 mm around stenosis part of PA because no tissue deformations appeared in other regions in the repair surgery. All experiments were performed on a NVIDIA Titan Xp (12 GB) GPU. Approximating running time was 1800 s.

**FIGURE 3:**
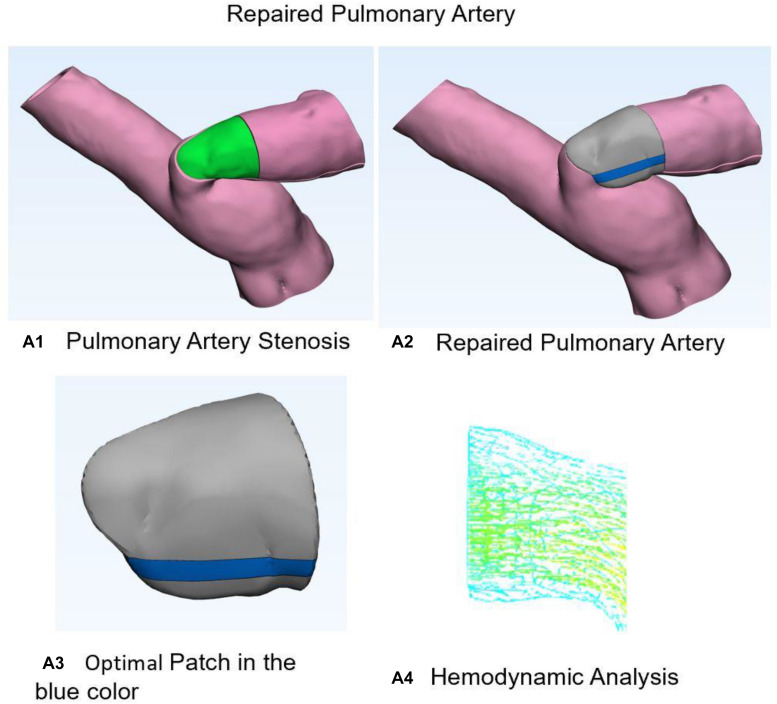
Stenosis and normal pulmonary artery. **(A1)** Three-dimensional anatomic model for pulmonary artery stenosis. Green area is the stenosis part. **(A2)** Shows the repaired pulmonary artery with optimal patch. **(A3)** Optimal patch in the blue color. **(A4)** Hemodynamic analysis for repair pulmonary artery with normal pressure and flow velocity.

Figure 3 shows the optimal repaired PA estimated from our model. Figure 3A1 demonstrates 3D anatomic model for PA stenosis. Green area is the stenosis part. Figure 3A2 shows the repaired PA with optimal patch. Figure 3A3 indicates optimal patch in the blue color. Figure 3A4 demonstrates hemodynamic analysis for repair PA with normal pressure of 30 mmHg and flow velocity of 1.2 m/s. All hemodynamic analyses were performed on ADINA software.

**FIGURE 4:**
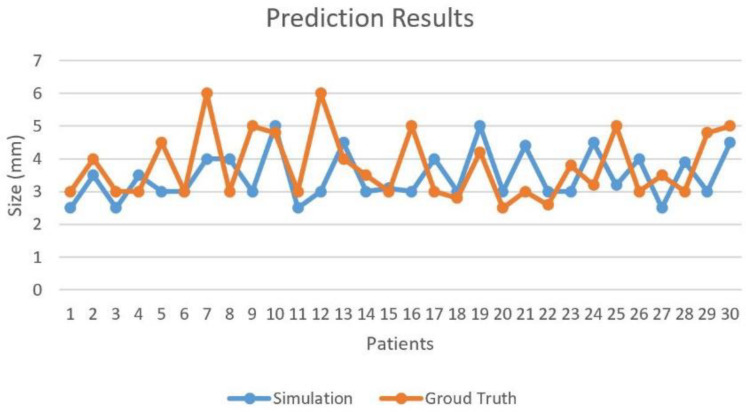
Prediction results for optimal patch design model.

Post-operative MRI of patients were considered as ground truth. Diameters of the repaired PA were measured and compared with our predicted model. Tolerance error of 2 mm was set to determine the correctness of our predicted patch. The fivefold CV was performed on 30 cases with ground truth for optimal patch prediction based on GANs in the repair of TOF and the CV accuracy was 93.33%. In Figure 4, Case **#7** and **#12** failed because both main PA and left PA were repaired. More factors were taken into account in real practice situation, such as the angle of the two branches of PA. Blood flow obstruction might also occur when this angle is too small.

## Discussion

**FIGURE 5:**
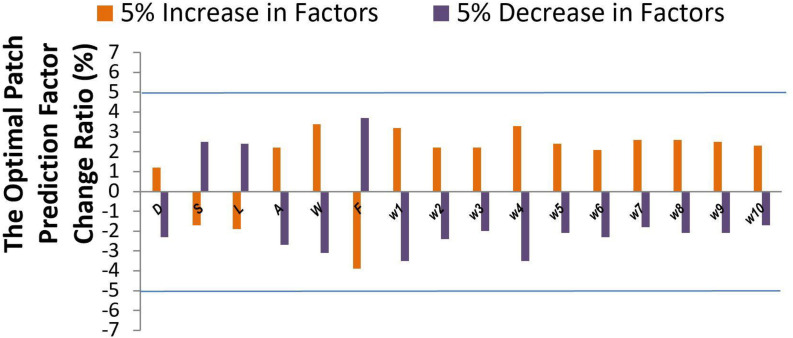
Sensitivity analysis.

Sensitivity analysis (Li et al., 2013) was performed to explore the model output variation upon perturbation of variables (Iman et al., 1981; Saltelli et al., 2000) such as patch size *D*, shape *S*, location *L*, mesh related cross section area *A*, wall shear stress *W*, blood flow rate *F*, and parameters (Ji et al., 2017) such as 10 coefficients $\left\{w^{i}\right\}\,{}_{i=1}^{10}$ which represent the important features via DX score (Ji et al., 2019; Hu et al., 2020). While a perturbation over a range of 5% was imposed on all factor values, the output variance was bounded by 5%, indicating the high stability of our model. The effectiveness of each factor was shown in Figure 5. Factors such as *D*, *S*, and *L* showed a low sensitivity (1.2–2.5 upon 5% parameter perturbation), while other factors such as *W* and *F* showed a higher sensitivity (3.1–3.9 upon 5% parameter perturbation) for optimal patch prediction. Our model can potentially underlie the mechanisms of TOF outcomes.

In this article, the results show that the proposed method is capable of substantial repair TOF in PA CT images, and that combining a voxel-wise squared error loss with adversarial loss led to an optimal patch generation that was similar to that in the reference normal PA CT image. This shows that the GANs model has a significant advantage in finding the best balance point of patch optimization training with only squared error loss led to repair images with stenosis part. Larger patch will cause outflow obstruction due to abnormal angle of main PA and left PA branches. Smaller patch cannot augment the stenosis artery to the normal shape. Thus, our optimal patch design system performed well by GANs with hemodynamic analysis for repair TOF. However, the patch deformation behaviors during diastolic and systolic of cardiac cycle were unable to be analyzed using the current system. Therefore, this system will be further improved by advanced biomechanical technology in the future.

As a result, this approach has the potential to reduce the intraoperative misjudgment rate, thereby providing a detailed surgical plan in patients with TOF.

## Data Availability Statement

The original contributions presented in the study are included in the article/supplementary material, further inquiries can be directed to the corresponding author/s.

## Ethics Statement

The retrospective study involving human participants was reviewed and approved by the Medical Research Ethics Committee of West China Hospital. The written informed consent of patients/participants were waived in this study.

## Author Contributions

YL: data collection. GZ and YM: data analysis and writing of the manuscript. YL, LP, and ML: data interpretation. XZ: research conception. GZ: critical revision of the manuscript. All authors reviewed and approved the manuscript.

## Conflict of Interest

The authors declare that the research was conducted in the absence of any commercial or financial relationships that could be construed as a potential conflict of interest.
